# Expression of Concern: Upregulation of microRNA-148a inhibits proliferation, invasion and migration while promoting apoptosis of cervical cancer cells by downregulating RRS1

**DOI:** 10.1042/BSR-20181815_EOC

**Published:** 2020-06-25

**Keywords:** Cervical cancer, microRNA-148a, RRS1, Proliferation, Invasion, Migration

The Editorial Office has been made aware of potential issues surrounding the scientific validity of this paper, hence has issued an expression of concern to notify readers whilst the Editorial Office investigates.

